# Lipid Nanocapsules for Imatinib Delivery: Design, Optimization and Evaluation of Anticancer Activity Against Melanoma Cell Line

**DOI:** 10.22037/ijpr.2019.1100870

**Published:** 2019

**Authors:** Mohammad Reza Molaahmadi, Jaleh Varshosaz, Somayeh Taymouri, Vajihe Akbari

**Affiliations:** a *Department of Pharmaceutics, School of Pharmacy, Isfahan University of Medical Sciences, Isfahan, Iran. *; b *Novel Drug Delivery Systems Research Centre, Isfahan University of Medical Sciences, Isfahan, Iran.*; c *Department of Pharmaceutical Biotechnology, School of Pharmacy, Isfahan University of Medical Sciences, Isfahan, Iran.*

**Keywords:** Cancer chemotherapy, Imatinib, Lipid nanocapsules, Phase-inversion temperature method, B16F10 melanoma cells

## Abstract

Lipid nanocapsules (LNCs) represent a stable, biocompatible and worthwhile drug delivery system, demonstrating significant potential as gene/drug delivery platforms for cancer therapy. Imatinib, a potent tyrosine kinase inhibitor, has revolutionized the therapy of malignancies resulting from abnormal tyrosine kinase activity. However, its Clinical effectiveness in cancer treatment is hampered by its off-target side effects. In this study, we have investigated the potential benefits of LNCs as a novel drug delivery vehicle for imatinib with a view to improve drug efficacy. LNC formulations were prepared by phase-inversion temperature method and the effects of various formulation variables were assessed using full factorial design. The cytotoxicity and cellular uptake of optimized formulation were investigated against B16F10 melanoma cell line. Analysis of result by Design-Expert® software indicated that Solutol HS15 percent was the most effective parameter on the encapsulation efficiency, particle size, zeta potential, and release efficiency of LNCs. The optimized formulation revealed a particle size of 38.96 ± 0.84 nm, encapsulation efficiency of 99.17 ± 0.086%, zeta potential of -21.5 ± 0.61 mV, release efficiency of 60.03 ± 4.29, and polydispersity index of 0.24 ± 0.02. The imatinib loaded LNCs showed no hemolysis activity. Fluorescent microscopy test showed that the cellular uptake of LNCs was time dependent and density of fluorescent signals increased with time in cells. The *in-vitro* cytotoxicity study indicated that imatinib kept its pharmacological activity when loaded into LNCs. These results introduced imatinib loaded LNCs as a promising candidate for further investigation in cancer therapy.

## Introduction

Cancer is a life threatening disease characterized by the uncontrolled proliferation and rapidly spreading of abnormal cells. Current strategies for cancer therapy include surgery, radiation, hormone therapy, and chemotherapy. Chemotherapy as the most common strategy in clinic, have improved patients survival; however because of non-specific distribution of the drug, both cancerous and normal cells are affected. This provides immoderate toxicities to normal cells while limiting the therapeutic dose within the cancer cells (1, 2). Nanotechnology is a promising approach in cancer treatment over the last decade based on the concept that nanoparticles (NPs) have the ability to carry loaded therapeutic agents to the cancer cells selectively by utilizing the unique pathophysiology of tumors with compromised leaky vasculature (3-5). Nanoparticulate drug delivery systems offer a number of advantages such as increasing solubility of hydrophobic drug, prolonging circulation time, improving drug release kinetic, minimizing non-specific uptake, improving intracellular penetration and allowing for specific cancer targeting (5). Among different types of NPs, lipid nano capsules (LNCs) have been extensively studied as promising carriers for hydrophobic drugs such as etoposide, paclitaxel, and tamoxifen as well as hydrophilic drugs like nucleic acids, and amphiphilic compounds (6-10). LNCs are lipoprotein like NPs composed of a liquid oily core and semi rigid shell (11). This shell made up of solutol^®^ HS15 and lecithin in its outer and inner parts, respectively. They have nano-scale dimensions in the range of 20-100 nm with sustained release behavior, prolonged circulation time, and increased tumor localization by enhanced permeation and retention effect (EPR) (12). In this context, Hureux *et al.* developed paclitaxel loaded LNCs to improve therapeutic index of paclitaxel (13). Paclitaxel loaded LNCs showed no histological and biochemical abnormalities in mice after a five day *iv* injection The *in-vivo *study in nude mice bearing lung cancer showed that paclitaxel loaded LNCs had superior antitumor activity compared to Taxol. Some other advantages of LNCs are simple and low cost preparation method by low energy phase inversion technique without using high quantity of surfactant and co-surfactant (11, 14), increasing the stability of the entrapped drugs, capability to inhibit the P-gp efflux pumps (12), low toxicity, good biocompatibility (15) and easy to scale up (16, 17). LNCs also have higher drug loading capacity and longer physical stability (up to 18 months) compared with liposomes and/or solid lipid nanoparticles (SLNs) (18). Imatinib, small tyrosine kinase molecules, has been reported to inhibit tyrosine-kinase activity of platelet derived growth factor receptor (PDGFR), c-kit, and bcr-able fusion protein (19, 20). Imatinib has demonstrated anticancer effect against chronic myeloid leukemia (CML), gastrointestinal stromal tumors (GIST), and other related diseases resulting from abnormal tyrosine kinase activity such as prostate cancer, colon cancer, cervical, and melanoma (19-24). Although bcr-able fusion protein is just expressed in cancer cells, c-kit and PDGFR receptors exist in healthy cells too and their inhibition by imatinib results toxicity such as unanticipated cardiotoxicity (19, 20). Other difficulties, such as variable systemic availability, development of resistance on long term due to activation of efflux transport system, and adverse effects such as myelo-suppression, reduction in red blood cell count, oedema, local gastric irritation, dyspepsia, skin rashes, and muscular cramps also hampered its clinical use (25, 26). In order to overcome these problems, nanoparticulate systems can be used as effective delivery tools to enhance the antitumor activity and decrease harmful side effects (27, 28). For instance, Kamali *et al.* reported that human serum albumin (HAS) NPs containing imatinib increased the cytotoxic effect of drug relative to free imatinib for glioblastoma (29). Poly(lactide-*co*-glycolide) NPs also increased the efficacy of imatinib against MCF-7 cells and decreased imatinib induced cardiotoxicity in wistar rats (20). Considering the advantages of LNCs for use in cancer therapy, in this study for the first time, we developed, optimized and characterized LNCs containing imatinib. After full characterization, to evaluate the potential of prepared lipid nanocapsules as drug delivery vehicles, cellular uptake of optimized formulation and its *in-vitro* cytotoxicity were studied against B16F10 melanoma cells.

## Experimental

*Materials*

Imatinib base was kindly provided by Parsian Pharmaceutical Co. (Iran). Labrafac (capric and caprylic acid triglyceride) was provided by BASF (Ludwigshafen, Germany). Solutol HS 15, coumarin 6 and soybean lecithin and dialysis bag (molecular cut off 12000 Da) were purchased from Sigma (USA). For cell culture study, B16F10 cell line was kindly provided by Pasteur Institute (Iran). 3-[4,5-dimethylthiazol-2-yl]-2,5-diphenyltetrazolium bromide (MTT) was purchased from Sigma Company (USA). Trypsin, fetal bovine serum (FBS), phosphate buffer saline (PBS), Dulbecco’s Modified Eagle Medium (DMEM), penicillin, and streptomycin were sourced from Gibco Laboratories (USA). 

*Preparation of imatinib loaded LNCs*

LNCs were prepared using phase-inversion temperature method. To prepare 2 g of LNCs, 20 mg of imatinib was dissolved in 300 µL of chloroform and then added to the labrafac solution (10-20% W/W) containing lecithin (1.5% W/W) as stabilizing agent under magnetic stirrer. The chloroform was then evaporated at 50 °C and the aqueous phase containing 1.75% (w/w) NaCl and different amounts of solutol HS 15 (15-30% w/w) was added. Finally, the mixture was subjected to three repeated cycles of heating and cooling from 85 °C to 60 °C. During the last cooling phase, an irreversible shock was induced by instant dilution by cold deionized water (0 °C). The fast-cooling dilution process led to breaking of microemulsion system and the formation of stable LNCs. Afterwards, the nanosuspension was stirred under slow magnetic stirring for 5 min (6, 30). 

To optimize conditions of the technical procedure, the full factorial design was used by Design-Expert® Software (version 10, US). Three different variables, including the amount percent of solutol HS 15 and labrafac as well as the volume ratio of the diluting aqueous phase to the initial emulsion (D/I ratio) were studied each in two levels to obtain eight different formulations (Tables 1 and 2). The evaluated responses were particle size, polydispersity index (PdI), zeta potential, encapsulation efficiency (EE%), and drug release efficiency during 48 h (RE_48_%). Dependent parameters were analyzed using Design-Expert® software and cutoff for significance of each factor was done by Analysis of variance (ANOVA).

*Particle size, PdI and zeta potential measurement*

The Particle size, PdI, and zeta potential of LNCs were measured by zeta sizer (PCS, Zeta sizer 3000, Malvern, UK). A dispersion of LNCs was diluted 30 times by deionized water at 25 ^o^C before analysis. Each test was done in triplicate. 

*Imatinib encapsulation efficiency (EE)*

For calculation of imatinib EE, 0.5 mL of each LNCs formulation was placed in microcentrifuge filter tube (Amicon Ultra, Ireland, cut off 10 kDa) and centrifuged (Sigma 3K30, Germany) at 14000 rpm for 10 min. The UV absorbance of imatinib in supernatant was then determined using UV spectrophotometer at 268 nm. EE was determined using the following Equations:

Equation 1 $$\mathrm{EE}=\left(\frac{\mathrm{total amount of drug added}-\mathrm{free drug}}{\mathrm{total amount of drug added}}\right)\mathrm{˟ 100}$$

*In-vitro release of imatinib from LNCs*

Release of imatinib from LNCs was assessed by dialysis method. One milliliter of each formulation was filled in the dialysis bag (cut-off 12000 Da) and then, the bag was placed in a glass tube containing appropriate amount of PBS with 0.5% tween 80 at 37 °C. One milliliter of release medium was taken away at predetermined time intervals up to 48 h, and refreshed with new medium. Then the content of the drug in the samples was determined using a UV spectrophotometer at 258 nm. To compare the release profile of different formulations, the RE_48_% was calculated by Equation 2:

Equation 2 $$\mathrm{RE}48\%=\frac{\int_{0}^{t}\mathrm{y.dt}}{\mathrm{y100.t}}\times 100$$

Where y is the released percent at time t.

*Morphological studies*

Scanning electron microscopy (SEM Hitachi F41100, Japan) was employed to observe morphology of the optimized LNCs. The LNCs dispersion was mounted on aluminum slabs, sputter-coated with a thin layer of Au/Pd and then scanned by the SEM.

*Study the kinetic of imatinib release*

The Imatinib release data obtained from the optimized formulations were fitted with the following kinetic models. 

Baker-Lonsdale: [1 - (1 - Q_t_/Q_∞_)^2/3^] - Q_t_/Q_∞_ = kt                              Equation 3

Hixson Crowell: (Q_0_^1/3 ^- Q^1/3 ^= kt)                               Equation 4

First order: (Ln (1 - Qt/Q∞) = -kt)                               Equation 5

Zero order: (Qt/Q∞ = kt)                               Equation 6

Higuchi: (Qt/Q∞ = kt^1/2^)                               Equation 7

In these equations, Q_0_ is the initial amount of the drug in NPs, Q_t_ is the amount of the drug released at time t, Q is the remaining amount of the drug in system, k is the release constant and Q_∞_ is the total amount of the drug loaded in LNCs intended to be released after infinite time. Correlation coefficient (R^2^) was used as an indicator of the best fitting of the model. The mechanism of the drug release from the prepared optimized NPs was also evaluated using Korsmeyer-Peppas equation (Q_t_/Q∞ = kt^n^, Equation 8) where n is the exponent parameter indicating to describe different release mechanisms of the drug. For diffusion controlled systems, n value is equal or less than 0.5 (0.45). When 0.5 < n < 1, diffusion- erosion is the dominant mechanism of the drug release. If n value is close to 1, the drug release is mainly controlled by erosion (relaxation) mechanism (31).

*Fourier-transform infrared spectroscopy (FTIR) analysis*

FTIR (Rayleigh, WQF-510/ 520, China) was used to evaluate any possible interaction between imatinib and different components of nanoparticulate system. The FTIR spectra scanned in the IR range from 400 to 4000 cm^-1^ using KBr pellet method.

*Hemolysis assay*

To evaluate the *in-vitro* hemocompatibility of the imatinib loaded LNCs, the fresh rat blood was centrifuged at 3000 rpm for 10 min. The settled red blood cells (RBCs) were separated and washed thrice with 0.9% normal saline to remove debris and serum protein. The stock of RBCs was prepared by mixing 2 mL of settled RBCs into 98 mL of normal saline 0.9% w/v. A predetermined amount of freshly prepared imatinib entrapping LNCs was mixed with RBCs suspension to give final imatinib concentration in a range from 5 to 50 μg/mL. Then, the samples were incubated at 37 °C for 1 h in a shaker incubator. 

The samples were withdrawn and centrifuged at 1500 rpm for 10 min to remove intact RBC and also the free hemoglobin in the supernatant was analyzed at 540 nm (32). To obtain 0 and 100% hemolysis, RBCs were mixed with 0.9% normal saline solution and distilled water as negative and positive control, respectively. The percentage of the hemolysis was measured by the following Equation:

 Hemolysis% = (ABS − ABS_0_/ABS_100_ − ABS_0_) × 100                              Equation 9

where ABS, ABS_0_, and ABS_100_ were denoted as the absorbance of formulation treated sample, a solution of 0% hemolysis, and a solution of 100% hemolysis, respectively. All hemolysis tests were carried out in triplicate. 

*Freeze-drying*

The freeze-drying process was carried out with 3% w/v sucrose, lactose and sorbitol as the cryoprotectants. Briefly, 2 mL of optimized LNCs containing imatinib was poured into glass vial and then an appropriate amount of sucrose, lactose, or sorbitol were added. 

Each glass vial was vortexed, frozen at −20 °C for 24 h and then lyophilized using freeze-dryer (Christ, Alpha 2-4 LD plus, Germany) at -80 °C under 0.4 bar for 48 h. The final samples were stored at 4 °C until analysis. After freeze drying, the products were reconstituted by the addition of original volume of distilled water to maintain drug and nanoparticle concentration. Then, the mean particle diameter, PdI, and zeta potential of the reconstituted products was determined by zeta sizer as described in the previous section.

*Cell viability assays*

B16F10 melanoma cell line was used to elucidate the cytotoxicity of imatinib-loaded LNCs by standard MTT assay. Briefly, B16F10 melanoma cells were seeded into 96-well plates at a density of 5 × 10^3^ cells per well and incubated for 24 h at 37 °C under 5% CO_2_ atmosphere. After that, the free imatinib, drug free LNCs, and imatinib loaded LNCs containing different imatinib concentrations were added, and the cells were incubated for additional 24 and 48 h. At the end of the experiments, 20 µL of MTT solution (5.0 mg/mL) was added into each well and incubated for another 4 h. Then, the culture medium was removed, and 150 µL of DMSO was added into each well to dissolve the formazan dyes. 

The absorbance of formazan was determined using a micro plate reader at 570 nm. The untreated cells were used as the control and the blank culture medium was used as a blank control. The Cell viability (%) was calculated based on the following Equation:

Cell viability%=Mean absorbance of sample- mean absorbance of blank Mean absorbance of control-mean absorbance of blank×100

                              Equation 10

*Cellular uptake*

The LNCs were loaded with coumarin 6 as a lipophilic fluorescent probe marker in a same way as imatinib LNCs, except 20 mg of imatinib, replaced with 2 mg of the probe. The cellular internalization of C6 loaded LNCs was investigated in B16F10 cells using fluorescent microscope (CETI, Belgium). The cells were seeded in 96-well plates at 5 × 10^3^ cells per well and incubated for 24 h to permit the cells attached. Then, the C6 loaded LNCs at concentrations of 2 µg/mL were added to the cells and incubated for another 1 and 3 h. At pre­determined intervals, the cells were washed with PBS and observed using a fluorescent microscope. 

*Statistical analysis*

Comparison between two groups was performed by the Student’s ***t***-test. All data is presented as mean ± SD, ***p*-**values of <0.05 was considered statistically significant.

## Results

In the present study, imatinib was loaded in LNCs by phase-inversion temperature method and its physicochemical properties were characterized. 

**Figure 1:**
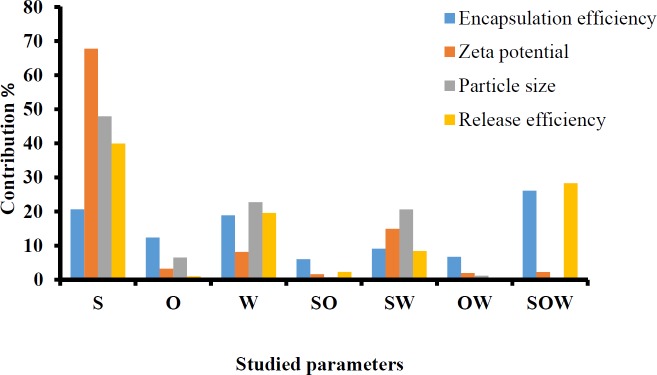
Contribution effect of different studied parameters on the particle size, zeta potential, encapsulation efficiency, RE48%. S: Surfactant%; O: Oil phase%; W: Volume ratio of the diluting aqueous phase to the initial emulsion

The full factorial design was employed to determine the optimum levels of independent variables including solutol HS 15 (%), labrafac (%) and D/I ratio. A summary of the physicochemical properties of imatinib loaded LNCs are presented in Table 3. The contribution effect of each independent variable on different responses including particle size, zeta potential, EE, and RE of imatinib loaded LNCS are shown in Figure 1.

*EE of imatinib in LNCs*

As shown in Table 3, EE in all investigated formulations were high and in the 97-99% range. As it can be seen from Figure 1, the most effective parameter on the EE was the solutol HS15 content. It was observed that by increasing the solutol HS 15 content, although not significant, the EE decreased (*P* > 0.05). 

*Particle size of LNCs*

**Figure 2:**
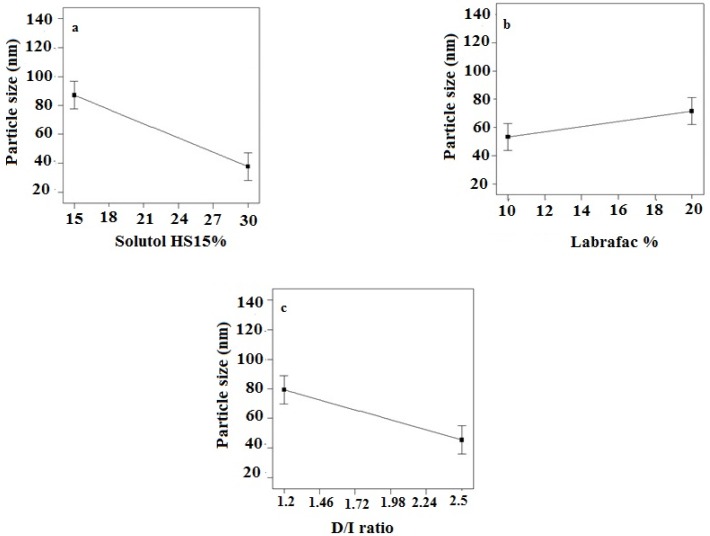
Effect of different levels of (a) solutol HS 15 (b) labrafac and (c) D/I ratio on the particle size of imatinib loaded LNCs

According to the data shown in Table 3, the particle size of LNCs for different formulations was in the range from 24 to 130 nm. The PdI, an indicator of homogeneity of nanosuspensions, fluctuated between minimum 0.16 and maximum 0.34 indicating a relatively narrow size distribution for prepared LNCs. Design of experiment (DOE) revealed that none of the studied parameters changed the PdI of LNCs significantly. Statistical analysis showed that particle size is more affected by content of solutol HS15. Effect of different levels of parameters on the particle size of imatinib-loaded LNCs is shown in Figure 2. It is concluded from ANOVA results that solutol HS15 content, labrafac content, D/I ratio, and interaction between solutol HS15 content and D/I ratio have significant effect on particle size of LNCs.

*Zeta potential*

**Figure 3.**
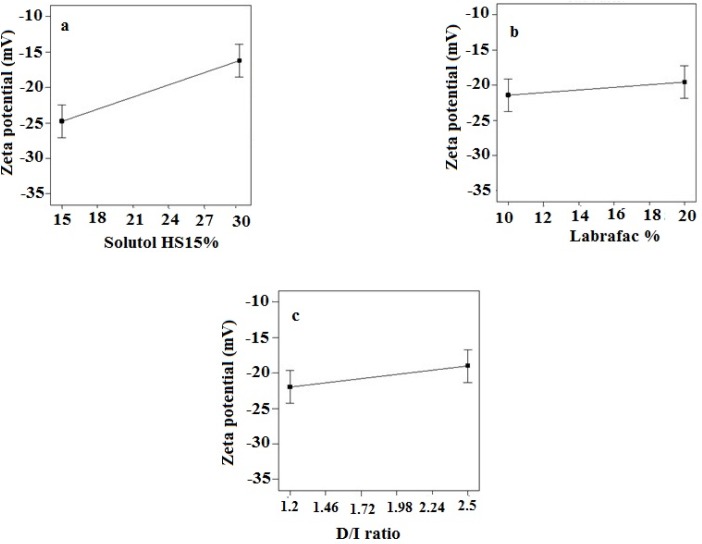
Effect of different levels of (a) solutol HS 15 (b) labrafac and (c) D/I ratio on the zeta potential of imatinib loaded LNCs

Zeta potential of the prepared LNCs ranged from -13.63 to -28.6 mV. The analysis of result by Design-Expert® software indicated that solutol HS15 content was the most effective factor on zeta potential. Furthermore, the ANOVA showed that solutol HS15 content and the interaction between solutol HS15 content and D/I ratio had significant influence on zeta potential of LNCs. Figure 3 shows the effect of each factor on zeta potential of imatinib loaded LNCs.

*In-vitro release of imatinib from LNCs*

**Figure 4.**
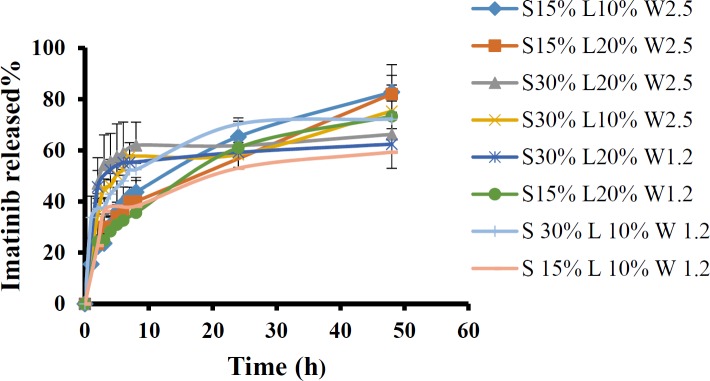
The release profiles of imatinib from various LNCs formulations

The release profiles of imatinib from various LNCs formulations are shown in Figure 4. All formulations showed biphasic release pattern with an initial burst followed by a more sustained release for 48 h.

**Figure 5:**
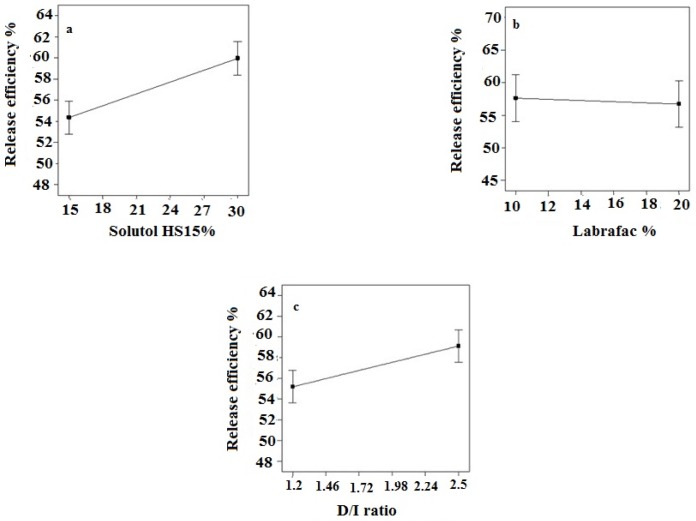
Effect of different levels of (a) solutol HS 15 (b) labrafac and (c) D/I ratio on the release efficiency of imatinib loaded LNCs

**Figure 6:**
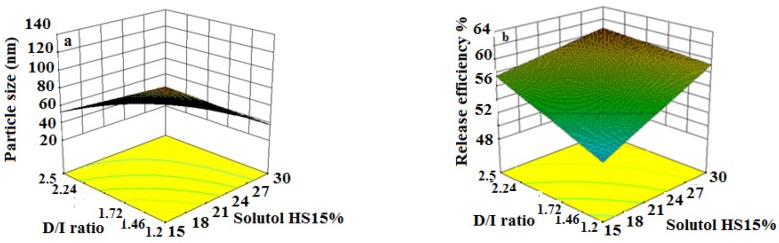
The effect of different level of D/I ratio - solutol HS15% on particle size and release efficiency of imatinib loaded LNCs.

The obtained RE_48_% is shown in Table 3. The solutol HS15 content was the most effectual factor on RE_48_% (Figure 1). Effect of different levels of parameters on the RE_48_% of imatinib-loaded LNCs is shown in Figure 5. It is concluded from ANOVA results that the content of solutol HS15 and the D/I ratio and their interaction have significant effect on RE_48_%. As shown in the 3-D response surface plots at constant content of labrafac, the highest RE_48_% and lowest particle size happen at high level of solutol HS15 content along with high D/I ratio (Figure 6).

*Optimization procedure*

**Figure 7:**
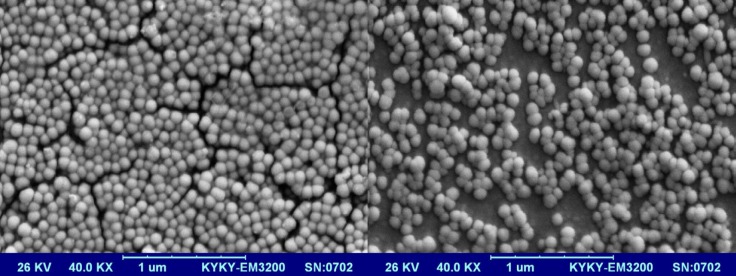
SEM images of imatinib loaded optimized LNCs

Optimization was done using Design-Expert® software and formulation coded S_15_O_10_W_2.5_ was selected as the most desirable formulation which showed size of 38.96 nm, PdI of 0.24, EE of 99.17%, RE48 of 60.03%, and zeta potential of -21.5 mV. Imatinib release from optimized LNCs was kinetically studied by Higuchi, zero order, first order, Baker-Lonsdale, and Hixson Crowell models. Table 4 shows correlation coefficient (R^2^) obtained from curve fitting of imatinib release data from the optimized LNC. Based on the highest correlation coefficient, it is suggested that the Baker-Lonsdale was the best fitted model. The value of n determined for LNCs from Korsmeyer-Peppas model was 0.4385 indicating that release phenomenon in optimized LNCs formulation is mainly governed by Fickian diffusion mechanism. SEM studies demonstrated that LNCs were in spherical shape and their particle size were close to the results obtained from DLS (Figure 7).

*FTIR study*

**Figure 8:**
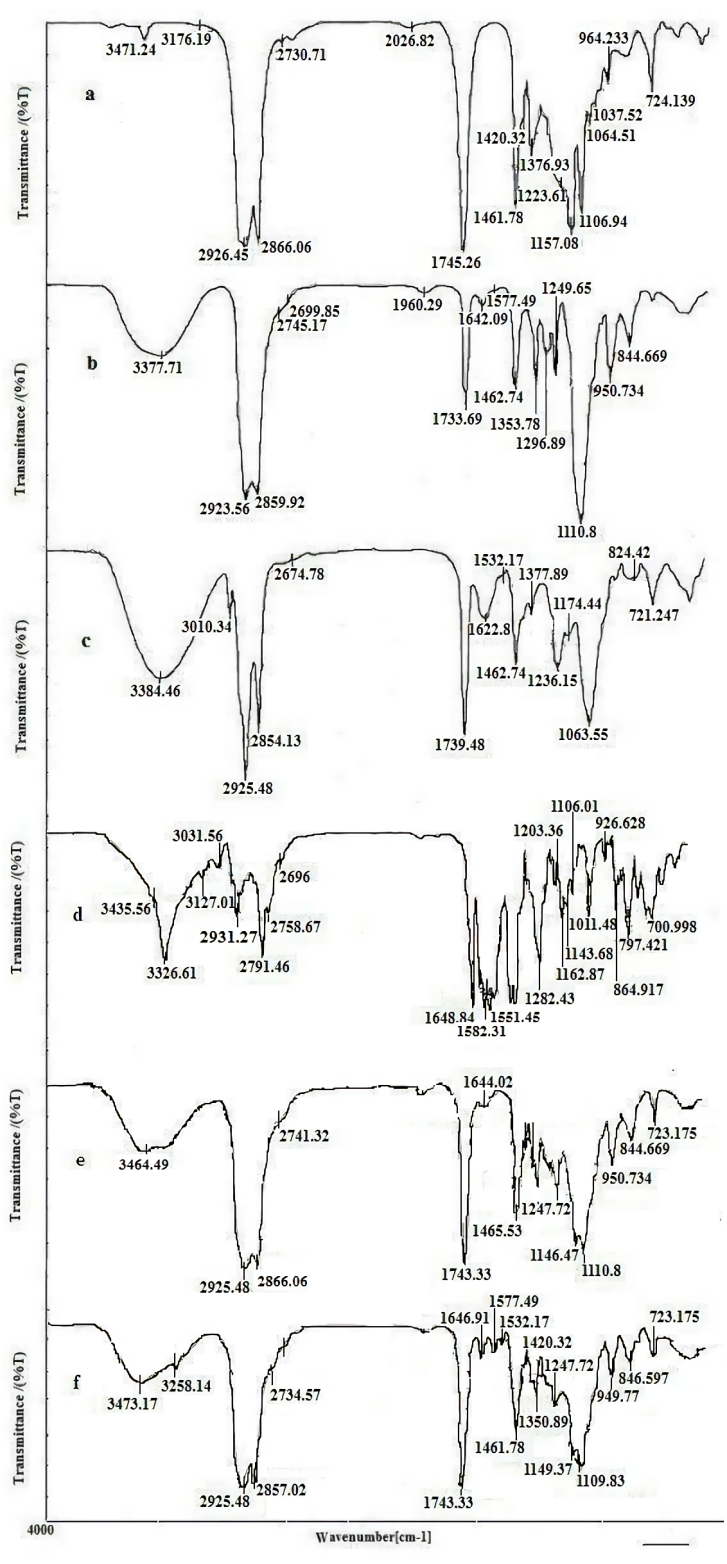
FTIR spectra of (a) labrafac, (b) solutol HS15, (c) lecithin, (d) imatinib, (e) blank LNCs (f) imatinib loaded LNCs

The compatibility between the drug and all ingredients used for preparation of LNCs were studied by FTIR spectroscopy. FTIR spectra of imatinib, solutol HS15, labrafac, lecithin, blank LNCs, and imatinib loaded LNCs are shown in Figure 8. The spectrum of imatinib displays characteristic bands at 3326.61 cm^-1^ (N-H stretch), 2931.27 cm^-1^ (C-H stretch, aromatic), 2791.46 cm^-1 ^(C-H stretch, aliphatic), 1648.84 cm^-1^ (C=O carbonyl), 1582 cm^-1^(C=C aromatic) and 1551 cm^-1^(C=N aromatic). The characteristic bonds observed from the IR spectra of solutol HS15 include the O-H bond at 3371.71 cm^-1^, ester group at 1733.69 cm^-1^. Labrafac showed absorption peaks of O-H stretch band at 3471.24 cm^-1^, aliphatic C–H bonds at 2856.06 and 2926.55 cm^−1^, C=O ester groups at 1745.26 cm^−1 ^and C-O groups at 1106.94 cm^−1^. Blank LNCs revealed O-H stretch band at 3464.49 cm^-1^, aliphatic C–H bonds at 2866, and 2925 cm^−1^, ester groups at 1743.33 cm^−1^ and C-O band at 1110 cm^−1^. The spectrum of imatinib loaded LNCs showed all characteristic bands were identified in the IR spectra of blank LNCs as well as the characteristics peaks of the drug with a little shift at 3285 cm^-1 ^(N-H stretch), 1646.91 cm^-1^ (C=O carbonyl), 1577.49 cm^-1^ (C=C aromatic), and 1532 cm^-1^(C=N).

*Hemolysis assay*

**Figure 9:**
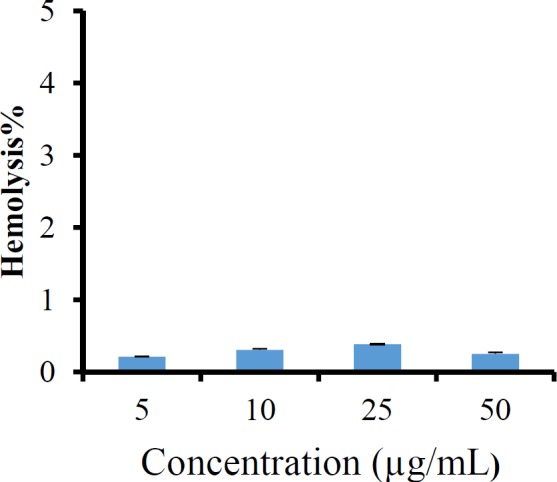
The percentage of hemolysis from imatinib loaded LNCs

Figure 9 displays the hemolysis activity of imatinib loaded LNCs. The results showed a very low hemolytic activity of LNCs at different concentrations of imatinib, indicating the hemocompatiblity of imatinib loaded LNCs.

*Freeze drying*

Considering the accelerated degradation of various types of polymers and lipids in water, the conversion of drug NP suspensions and emulsions into solid powder is important. Freeze-drying is one of most attractive industrial process to ensure high stability, ease in storage, and handling of colloidal system. In a first set of the experiments, imatinib loaded optimized LNCs were freeze dried without the usages of cryoprotectant. However, after freeze-drying, the resultant powder turned out to be sticky form and its redispersion into a suspension form was not feasible. 

Therefore, the freeze-drying process was carried out with 3% w/v sucrose, lactose, and sorbitol as the cryoprotectants. Table 5 shows the particle size, PdI and zeta potential of freeze-dried imatinib loaded LNCs after reconstitution. The freeze-dried product obtained by utilizing sorbitol formed a sticky collapsed cake. However, lactose and sucrose as cryoprotectant produced intact fluffy powder easily reconstituted to form LNCs in less than 10 sec. Sf/Si (ratio of particle size after and before freeze drying) for the sucrose and lactose was observed to be 3.28 and 1.23, respectively.

*Cell viability assays*

**Figure 10:**
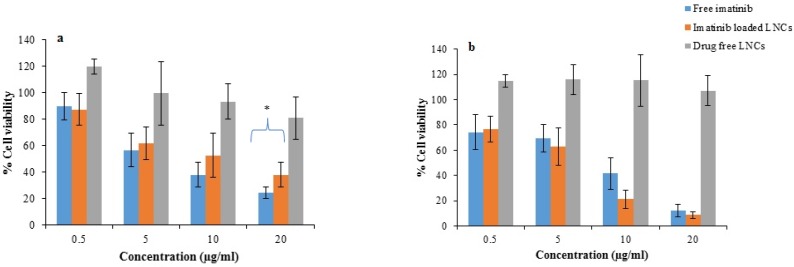
Cell viability (a) after 24 and (b) 48 h treatment with free imatinib, imatinib loaded LNCs and drug free LNCs. (mean ± SD, n = 6), ^*^p < 0.05 *vs.* free drug

Figure 10 represents the cell viability after 24 and 48 h treatment with free imatinib, imatinib loaded LNCs, and drug free LNCs. As shown, free imatinib and imatinib loaded LNCs showed a dose-dependent decrease in the viability of B16F10 cells. The results revealed that the IC_50_ of free imatinib was 6.5 µg/mL, which is lower than that of imatinib loaded LNCs (8.3 µg/mL) for 24 h incubation whereas, after 48 h, the IC_50_ for free imatinib and imatinib loaded LNCs were 6.5 µg/mL and 6.1 µg/mL, respectively.

*Cellular uptake study*

**Figure 11:**
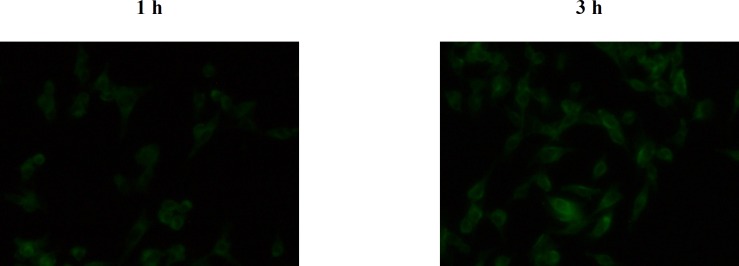
Fluorescent microscopic images of B16F10 cells after 1 and 3 h incubation at 37 °C with C6 loaded LNCs.

The insoluble dye Coumarin-6, as a fluorescence marker, has been widely used to replace the drug in the NPs and to measure the cellular uptake of NPs thanks to several unique features such as its biocompatibility, high fluorescence activity, and low leaky rate (33). Figure 11 represents fluorescent microscopy images of B16F10 cell line after 1 and 3 h incubation with the C6 loaded LNCs. The green fluorescence coming from Coumarin-6 loaded LNCs was seen within B16F10 cells which is due to internalization of the LNCs in the cells. The results also showed that the cellular uptake of LNCs was time dependent and density of fluorescent signals increased with time in B16F10 cells demonstrating more LNCs entered the cells.

## Discussion

LNCs containing drug is generally prepared by PIT method. This technique is based on the ability of emulsion to undergo a phase inversion from an o/w to a w/o or vice versa following a variation of temperature. This is due to presence of polyethoxylated nonionic surfactant such as solutol HS15 that their affinity for different phases or in other words, their hydrophilic/ lipophilic balance changed with temperature. This change occurred in phase inversion zone (PIZ). A formulation process consistent of two steps: Step I: Magnetic stirring of all the components and heating from room temperature to 85 °C (temperature greater than PIZ) to obtain W/O emulsion. This is followed by progressive cooling from 85 °C to 60 °C (temperature below PIZ) to obtain an O/W emulsion. Three temperature cycles (85–60–85–60–85 °C) were carried out. Step II is an irreversible shock induced by instant dilution by cold deionized water (0 °C) at the temperature which set at 1–3 °C from the beginning phase inversion zone of o/w emulsion (72 °C). The fast-cooling dilution process led to breaking system and the formation of stable LNCs with crystallized shell. Then, the nanosuspension was stirred under slow magnetic stirring for 5 min (15, 18). Previous studies showed that formulation results were a function of nature and ratio of various excipients employed for preparation (11). To obtain formulation with desired outcomes, optimum level of each mentioned variable was investigated. 

The present study showed that the EE in all investigated formulation was high and did not undergo significant change from one formulation to another. The possible reason may be due to the lipophilic nature and low solubility of imatinib base in the external aqueous phase as well as lipophilic nature of nanocarrier matrices. It was observed that by increasing the solutol HS 15 content, the EE decreased (*P *> 0.05). This finding could be attributed to the increased solubility of the drug in the aqueous phase as the percentage of surfactant increased (34). Particle size is one of the most important characteristics of the NPs, affecting the kinetic of release, cellular uptake pathway of NPs, and biodistribution (27). The decrease in the particle size of LNCs in higher amount of solutol HS15 (*P *< 0.05) is attributed to the smaller volume of the oil phase droplets and the greater reduction in the interfacial tension due to insertion of more amount of solutol HS15 into the interface (9, 11). This protects the droplets from aggregation and resulting consequently smaller LNCs at higher solutol HS15 concentrations. The findings were in agreement with previous report of Barras *et al.* who showed a reduction in the particle size of polyphenol-loaded LNCs with increasing solutol HS 15 content (35). An increment of labrafac in the mixture led to enlargement of core size of LNCs which in turn caused an increase of the mean particle size of LNCs (Figure 2b). In a parallel line, Heurtault *et al.* found that increase in the oil content of LNCs resulted in formation of LNCs with larger size (11). In the final stage of preparation of LNCs, an irreversible shock induced in system by fast cooling with the cold water to break the system and create nano objects (36). As shown in Figure 2c, increasing the volume of diluting cold water also decreased particle size thanks to inhibition of droplets aggregation with large amount of aqueous water available (*p* < 0.05). Zeta potential is charge at surface of NPs in solution and its magnitude is a key factor to determine the stability of NPs. The zeta potential of all the prepared LNCs was negative which may be attributed to the presence of negatively charged phospholipids at capsule interface (Table 3). Increasing solutol HS15 concentration decreased the absolute value of zeta potential (Figure 3a). Zeta potential is a function of surface coverage by charged species at a specified pH. The zeta potential value changes with the adsorption of surfactant on the surface of the particles. Considering the nanionic nature of solutol HS15, the covering layer of surfactant might shield the negative charge of phospholipids at capsule interface (9, 37). An increment of labrafac content, though not significant, augmented the absolute value of zeta potential which could be related to the increased contribution of negative phospholipids molecules integrated at capsule interface. The *in-vitro* drug release curve showed a biphasic pattern. The initial burst release might be related to immediate dissolution of the drug embedded near the particle surface and is beneficial for inhibiting cancer cell growth within the early hours of administration. After the initial burst release, drug diffuses through the lipid matrix into the release medium and generates a sustained release profile up to 48 h which enable the LNCs to continually suppress tumor cells (38). The RE_48_% parameter was employed for assessing drug release and used to compare release profiles. The greater RE_48_%, the faster the drug release rate will be. As it can be seen in Figure 5, the RE_48_% increased with decreasing the proportion of labrafac in the mixture as well as increasing solutol HS15 content and D/I ratio. All these changes decreased the particle size of the NPs which is usually reported to be a parameter influencing the release rate (39). By decreasing the NPs size, the surface area of the particles increases which causes an increase in the rate of drug release from NPs. This finding was confirmed by other studies including that of Taymouri and co-workers who reported that the decrease in particle size of nanomicelles increased the release rate of docetaxel (2). Increasing the volume of diluting phase also increased RE_48_%. This was in accordance with the previous study of Varshosaz *et al.* who showed an increase in RE_48_% with increasing the cold water volume (15). 

Computer optimization process by Design-Expert® software and a desirability function determined the effect of the levels of independent variables on the responses. Optimization for response factors includes minimum value of particle size, maximum absolute value of zeta potential, maximum value of RE_48_% and EE. The optimized formulation proposed by software with desirability factor equal to 74% was S_15_O_10_W_2.5_. This formulation was prepared by 10% labrafac, 15% solutol HS15 with D/I ratio of 2.5. Analysis of release data of optimized LNCs revealed that diffusion mechanisms play a main role in imatinib release from LNCs. The compatibility between drug and all ingredients used for preparation of LNCs were studied by FTIR spectroscopy. A Little shifting in peaks of drug, observed in spectrum of imatinib loaded LNCs could be attributed to intermolecular hydrogen bonding between the N-H group of the drug molecule and OH groups present in LNCs. The biocompatibility of the optimized formulation was investigated by hemolysis study. The result indicated that imatinib entrapping LNCs at different concentration of imatinib did not show any observational hemolytic activities in the RBC. Such low hemolytic activity with the developed formulation indicated good hemocompatibility. According to Guiding Principles of Hemolysis Test [H] GPT4-1, a hemolysis of less than 5% is regarded as non-hemolytic activity and therefore, imatinib loaded LNCs had no hemolytic effect on the RBC (40). Optimized EFV loaded LNCs were freeze dried using 3% w/v sucrose, lactose, and sorbitol as the cryoprotectants. From this result, lactose can maintain the size of NPs after rehydration. Therefore, on the basis of freeze dried cake properties, reconstitution behavior and DLS data after rehydration, it can be said that lactose is the most suitable cryoprotectant for imatinib entrapping LNCs.

The *in-vitro* cytotoxicities of imatinib loaded LNCs and free imatinib were evaluated by MTT assay in B16F10 cells. Drug free LNCs were also used at equivalent concentration of materials used for preparation of imatinib loaded LNCs. The result showed more than 80% of B16F10 cells remained viable after 24 and 48 h incubation with drug free LNCs demonstrating drug free LNCs are nontoxic. After 24 h, free imatinib showed a lower IC_50_ value (6.5 µg/mL) compared to that of imatinib loaded LNCs (8.3 µg/mL). This finding could be related to the simple diffusion of free imatinib into B16F10 cells and time consuming drug release from imatinib loaded LNCs, thereby leading to rapid effect of free imatinib on the cytotoxicity of cells. This data is consistent with the results in the other literatures employing NPs containing drug where free drug showed higher cytotoxicity than drug loaded NPs (41-48). The viabilities of B16F10 cells treated with imatinib loaded LNCs at 48 h were decreased in comparison with those at 24 h and the cell cytotoxicity of imatinib loaded LNCs was higher or at least similar to that of free imatinib at all concentration. The IC_50_ of B16F10 cells treated with imatinib loaded LNCs was 6.1 µg/mL and slightly lower than that of free imatinib group (6.5 µg/mL) after incubation for 48 h. In summary, the obtained findings revealed that the pharmacological activity of imatinib will be maintained when loaded into LNCs. The ability of LNCs for passive targeting to the tumor site and sustained release of the drug may have benefits in reduction of the need to the high doses of drug and consequently its side effects (46).

**Table 1:** Variables and their levels used in the full factorial design

**Variables**	**Levels**		**Dependent variables**
	**Ι**	**II**	
Surfactant% (S)	15%	30%	Particle size (nm)
Oil phase% (O)	10%	20%	Polydispersity index
Volume ratio of the diluting aqueous phase to the initial emulsion (W)	1.2	2.5	Zeta potential (mV) Encapsulation efficiency (%) Release efficiency (%)

**Table 2:** Composition of imatinib loaded LNCs.

**Formulations**	**Solutol HS 15**	**Labrafac (%)**	**Volume ratio of diluting aqueous phase to initial emulsion**
S15O10W2.5	15	10	2.5
S15O20W2.5	15	20	2.5
S30O20W2.5	30	20	2.5
S30O10W2.5	30	10	2.5
S30O20W1.2	30	20	1.2
S15O20W1.2	15	20	1.2
S30O10W1.2	30	10	1.2
S15O10W1.2	15	10	1.2

S: Surfactant%; O: Oil phase%; W: Volume ratio of the diluting aqueous phase to the initial emulsion.

**Table 3:** Physicochemical characteristics of imatinib loaded LNCs

**Formulations**	**Particle size (nm)**	**PdI**	**Zeta potential (mV)**	**Encapsulation efficiency (%)**	**Release efficiency (RE** **48** **%)**
S15O10W2.5	38.96 ± 0.84	0.24 ± 0.02	-21.5 ± 0.61	99.17 ± 0.086	60.03 ± 4.29
S15O20W2.5	68.66 ± 0.92	0.16 ± 0.05	-21.06 ± 0.89	98.65 ± 0.23	55.19 ± 6.18
S30O20W2.5	48.07 ± 0.26	0.29 ± 0.006	-13.63 ± 0.30	98.02 ± 0.43	61.6 ± 6.63
S30O10W2.5	25.44 ± 1.84	0.17 ± 0.02	-19.85 ± 1.54	99.34 ± 0.28	59.69 ± 3.36
S30O20W1.2	39.12 ± 0.64	0.19 ± 0.032	-15.6 ± 1.05	98.07 ± 0.28	56.09 ± 5.86
S15O20W1.2	30.2 ± 2.04	0.34 ± 0.037	-27.95 ± 2.1	98.07 ± 0.19	53.98 ± 7.36
S30O10W1.2	37.64 ± 5.32	0.23 ± 0.06	-15.77 ± 1.004	97.07 ± 0.105	62.49 ± 2.46
S15O10W1.2	110.92 ± 10.39	0.23 ± 0.02	-28.6 ± 2.007	99.35 ± 0.014	48.25 ± 6.5

S: Surfactant%; O: Oil phase%; W: Volume ratio of the diluting aqueous phase to the initial emulsion.

**Table 4:** Correlation coefficient (R2) obtained from curve fitting of imatinib release data from optimized LNC

**Formulation**	**Kinetic models (r2)**					**Korsmeier-Peppas parameters**	
	**Baker- Lonasdale**	**Higuchi**	**Hixon- Crowel**	**First order**	**Zero order**	**n**	**r** **2**
S15 O10W2.5	0.9975	0.9747	0.7454	0.9818	0.8802	0.4385	0.9793

S: Surfactant%; O: Oil phase%; W: Volume ratio of the diluting aqueous phase to the initial emulsion.

**Table 5:** Particle size, PDI and zeta potential of freeze-dried imatinib loaded LNCs

**Cryoprotectant**	**Particle size (nm)**	**PdI**	**Zeta potential (mV)**
Sorbitol	53.42 ± 0.54	0.35 ± 0.025	-40.9 ± 2.3
Lactose	47.91 ± 0.30	0.32 ± 0.008	-39.13 ± 0.90
Sucrose	127.93 ± 2.57	0.29 ± 0.02	-55 ± 2.57

## Conclusion

Imatinib loaded LNCs were successfully prepared by phase-inversion temperature method with satisfactory particle size, PdI, zeta potential, and EE. Full Factorial Design was utilized to optimize the formulation variables. The best results obtained from LNCs prepared by 10% labrafac, 15% solutol HS15 when the D/I ratio was 2.5. SEM studies demonstrated that the optimized LNCs were in spherical shape and monodispersed. Imatinib loaded LNCs were found to be hemocompatible. The cytotoxicity studies showed that the LNCs keep the biological activity of drugs. Finding of the present study showed that prepared LNCs could be an effective vehicle for imatinib delivery. However, further in depth *in-vivo* studies are required to evaluate the efficacy and side effects of formulation.

## References

[1] Nguyen KT (2011). Targeted nanoparticles for cancer therapy: promises and challenge. J. Nanomedic. Nanotechnol.

[2] Taymouri S, Varshosaz J, Hassanzadeh F, Javanmard SH, Dana N (2015). Optimisation of processing variables effective on self-assembly of folate targeted synpronic-based micelles for docetaxel delivery in melanoma cells. IET Nanobiotechnol.

[3] Haley B, Frenkel E (2008). Nanoparticles for drug delivery in cancer treatment. Urol. Oncol.

[4] Jabir NR, Tabrez S, Ashraf GM, Shakil S, Damanhouri GA, Kamal MA (2012). Nanotechnology-based approaches in anticancer research. Int. J. Nanomedicine.

[5] Taymouri S, Varshosaz J (2014). The recent progresses on the improved therapy of melanoma by novel drug delivery systems. Curr. Drug Targets.

[6] Allard E, Passirani C, Garcion E, Pigeon P, Vessières A, Jaouen G, Benoit JP (2008). Lipid nanocapsules loaded with an organometallic tamoxifen derivative as a novel drug-carrier system for experimental malignant gliomas. J. Control. Release.

[7] Peltier S, Oger JM, Lagarce F, Couet W, Benoît JP (2006). Enhanced oral paclitaxel bioavailability after administration of paclitaxel-loaded lipid nanocapsules. Pharm. Res.

[8] Lamprecht A, Benoit JP (2006). Etoposide nanocarriers suppress glioma cell growth by intracellular drug delivery and simultaneous P-glycoprotein inhibition. J. Control. Release.

[9] Lamprecht A, Bouligand Y, Benoit JP (2002). New lipid nanocapsules exhibit sustained release properties for amiodarone. J. Control. Release.

[10] David S, Resnier P, Guillot A, Pitard B, Benoit JP, Passirani C (2012). siRNA LNCs–a novel platform of lipid nanocapsules for systemic siRNA administration. Eur. J. Pharm. Biopharm..

[11] Heurtault B, Saulnier P, Pech B, Venier-Julienne MC, Proust JE, Phan-Tan-Luu R, Benoît JP (2003). The influence of lipid nanocapsule composition on their size distribution. Eur. J. Pharm. Sci.

[12] Safwat S, Hathout RM, Ishak RA, Mortada ND (2017). Augmented simvastatin cytotoxicity using optimized lipid nanocapsules: a potential for breast cancer treatment. J. Liposome Res.

[13] Hureaux J, Lagarce F, Gagnadoux F, Rousselet MC, Moal V, Urban T, Benoit JP (2010). Toxicological study and efficacy of blank and paclitaxel-loaded lipid nanocapsules after i administration in mice. Pharm. Res.

[14] Saliou B, Thomas O, Lautram N, Clavreul A, Hureaux J, Urban T, Benoit JP, Lagarce F (2013). Development and in-vitro evaluation of a novel lipid nanocapsule formulation of etoposide. Eur. J. Pharm. Sci.

[15] Varshosaz J, Hajhashemi V, Soltanzadeh S (2011). Lipid nanocapsule-based gels for enhancement of transdermal delivery of ketorolac tromethamine. J. Drug Deliv.

[16] Thomas O, Lagarce F (2013). Lipid nanocapsules: a nanocarrier suitable for scale-up process. J. Drug Deliv. Sci. Technol.

[17] Sánchez-Moreno P, Buzón P, Boulaiz H, Peula-García JM, Ortega-Vinuesa JL, Luque I, Salvati A, Marchal JA (2015). Balancing the effect of corona on therapeutic efficacy and macrophage uptake of lipid nanocapsules. Biomaterials.

[18] Huynh NT, Passirani C, Saulnier P, Benoit JP (2009). Lipid nanocapsules: a new platform for nanomedicine. Int. J. Pharm.

[19] Harata M, Soda Y, Tani K, Ooi J, Takizawa T, Chen M, Bai Y, Izawa K, Kobayashi S, Tomonari A, Nagamura F (2004). CD19-targeting liposomes containing imatinib efficiently kill Philadelphia chromosome–positive acute lymphoblastic leukemia cells. Blood.

[20] Marslin G, Revina AM, Khandelwal VK, Balakumar K, Prakash J, Franklin G, Sheeba CJ (2015). Delivery as nanoparticles reduces imatinib mesylate-induced cardiotoxicity and improves anticancer activity. Int. J. Nanomedicine.

[21] Deinlein T, Wolf IH, Rainer B, Kupsa R, Richtig E, Hofmann-Wellenhof R, Zalaudek I (2017). Treatment of primary and metastatic multifocal mucosal melanoma of the oral cavity with imatinib. CaseRep. Oncol..

[22] Mendonça LS, Moreira JN, de Lima MC, Simoes S (2010). Co‐encapsulation of anti‐BCR‐ABL siRNA and imatinib mesylate in transferrin receptor‐targeted sterically stabilized liposomes for chronic myeloid leukemia treatment. Biotechnol. Bioeng..

[23] Ye P, Zhang W, Tan Yang YL, Lu M, Gai Y, Ma X, Xiang G (2014). Folate receptor-targeted liposomes enhanced the antitumor potency of imatinib through the combination of active targeting and molecular targeting. Int. J. Nanomedicine.

[24] Danchev N, Nikolova I, Momekov G (2008). A new era in anticancer therapy/imatinib—a new era in anticancer therapy. Biotechnol. Biotec. Eq..

[25] Negi LM, Jaggi M, Joshi V, Ronodip K, Talegaonkar S (2015). Hyaluronan coated liposomes as the intravenous platform for delivery of imatinib mesylate in MDR colon cancer. ‎Int. J. Biol. Macromol..

[26] He C, Hu Y, Yin L, Tang C, Yin C (2010). Effects of particle size and surface charge on cellular uptake and biodistribution of polymeric nanoparticles. Biomaterials.

[27] Yadav KS, Jacob S, Sachdeva G, Chuttani K, Mishra AK, Sawant KK (2011). Long circulating PEGylated PLGA nanoparticles of cytarabine for targeting leukemia. J. Microencapsul..

[28] Kamali M, Dinarvand R, Maleki H, Arzani H, Mahdaviani P, Nekounam H, Adabi M, Khosravani M (2015). Preparation of imatinib base loaded human serum albumin for application in the treatment of glioblastoma. RSC Adv.

[29] Groo AC, Saulnier P, Gimel JC, Gravier J, Ailhas C, Benoit JP, Lagarce F (2013). Fate of paclitaxel lipid nanocapsules in intestinal mucus in view of their oral delivery. Int. J. Nanomedicine.

[30] Mashak A, Mobedi H, Mahdavi H (2015). A comparative study of progesterone and lidocaine hydrochloride release from poly (L-lactide) films. Pharm. Sci..

[31] Li Y, Qi XR, Maitani Y, Nagai T (2009). PEG–PLA diblock copolymer micelle-like nanoparticles as all-trans-retinoic acid carrier: in-vitro and in-vivo characterizations. Nanotechnology.

[32] Zhang Y, Tang L, Sun L, Bao J, Song C, Huang L, Liu K, Tian Y, Tian G, Li Z, Sun H (2010). A novel paclitaxel-loaded poly (ε-caprolactone)/poloxamer 188 blend nanoparticle overcoming multidrug resistance for cancer treatment. Acta Biomater.

[33] Emami J, Mohiti H, Hamishehkar H, Varshosaz J (2014). Formulation and optimization of solid lipid nanoparticle formulation for pulmonary delivery of budesonide using Taguchi and box-behnken design. Res. Pharm. Sci.

[34] Barras A, Mezzetti A, Richard A, Lazzaroni S, Roux S, Melnyk P, Betbeder D, Monfilliette-Dupont N (2009). Formulation and characterization of polyphenol-loaded lipid nanocapsules. Int. J. Pharm.

[35] Heurtault B, Saulnier P, Pech B, Proust JE, Benoit JP (2002). A novel phase inversion-based process for the preparation of lipid nanocarriers. Pharm. Res.

[36] Pourmashhadi A, Sadeghi H, Varshosaz J, Hamishehkar H (2015). Formulation and optimization of celecoxib-loaded PLGA nanoparticles by the Taguchi design and their in-vitro cytotoxicity for lung cancer therapy. Pharm. Dev. Technol.

[37] Zhai Y, Liu M, Wan M, Li Y, Zhang M, Zhai G (2015). Preparation and characterization of puerarin-loaded lipid nanocapsules. J. Nanosci. Nanotechnol.

[38] Varshosaz J, Taymouri S, Minaiyan M, Rastegarnasab F, Baradaran A (2018). Development and in-vitro/in-vivo evaluation of HPMC/chitosan gel containing simvastatin loaded self-assembled nanomicelles as a potent wound healing agent. Drug Dev. Ind. Pharm.

[39] Li X, Yang Z, Yang K, Zhou Y, Chen X, Zhang Y, Wang F, Liu Y, Ren L (2009). Self-assembled polymeric micellar nanoparticles as nanocarriers for poorly soluble anticancer drug ethaselen. Nanoscale Res. Lett.

[40] Lian H, Du Y, Chen X, Duan L, Gao G, Xiao C, Zhuang X (2017). Core cross-linked poly (ethylene glycol)-Graft-Dextran nanoparticles for reduction and pH dual responsive intracellular drug delivery. J. Colloid Interface Sci.

[41] Park S, Yoo HS (2010). In-vivo and in-vitro anti-cancer activities and enhanced cellular uptakes of EGF fragment decorated doxorubicin nano-aggregates. Int. J. Pharm.

[42] Liu H, Wu S, Yu J, Fan D, Ren J, Zhang L, Zhao J (2017). Reduction-sensitive micelles self-assembled from amphiphilic chondroitin sulfate A-deoxycholic acid conjugate for triggered release of doxorubicin. Mater. Sci. Eng. C.

[43] Liu Q, Li R, Zhu Z, Qian X, Guan W, Yu L, Yang M, Jiang X, Liu B (2012). Enhanced antitumor efficacy, biodistribution and penetration of docetaxel-loaded biodegradable nanoparticles. Int. J. Pharm.

[44] Wang X, Wang Y, Chen X, Wang J, Zhang X, Zhang Q (2009). NGR-modified micelles enhance their interaction with CD13-overexpressing tumor and endothelial cells. J. Control. Release.

[45] Seo DH, Jeong YI, Kim DG, Jang MJ, Jang MK, Nah JW (2009). Methotrexate-incorporated polymeric nanoparticles of methoxy poly (ethylene glycol)-grafted chitosan. Colloids Surf. B Biointerfaces.

[46] Zhang Z, Feng SS (2006). The drug encapsulation efficiency, in-vitro drug release, cellular uptake and cytotoxicity of paclitaxel-loaded poly (lactide)–tocopheryl polyethylene glycol succinate nanoparticles. Biomaterials.

[47] Sepehri N, Rouhani H, Tavassolian F, Montazeri H, Khoshayand MR, Ghahremani MH, Ostad SN, Atyabi F, Dinarvand R (2014). SN38 polymeric nanoparticles: in-vitro cytotoxicity and in-vivo antitumor efficacy in xenograft balb/c model with breast cancer versus irinotecan. Int. J. Pharm..

